# High levels of circulating interferons type I, type II and type III associate with distinct clinical features of active systemic lupus erythematosus

**DOI:** 10.1186/s13075-019-1878-y

**Published:** 2019-04-29

**Authors:** Vilija Oke, Iva Gunnarsson, Jessica Dorschner, Susanna Eketjäll, Agneta Zickert, Timothy B. Niewold, Elisabet Svenungsson

**Affiliations:** 1 Rheumatology Unit, Department of Medicine, Karolinska Institutet, Karolinska University Hospital, SE-171 76 Stockholm, Sweden; 2 Division of Rheumatology and Department of Immunology, Mayo Clinic, Rochester, MN USA; 3 Cardiovascular, Renal and Metabolism, IMED Biotech Unit, AstraZeneca, Integrated Cardio Metabolic Centre, Karolinska Institutet, Huddinge, Sweden; 4 Colton Center for Autoimmunity, New York University School of Medicine, New York, NY USA

**Keywords:** SLE, Interferons, Disease activity, Autoantibodies

## Abstract

**Background and aim:**

Interferons (IFNs) are considered to be key molecules in the pathogenesis of systemic lupus erythematosus (SLE). We measured levels of type I, II and III IFNs in a large cohort of patients with systemic lupus erythematosus (SLE) and controls and explored associations among high levels of different IFN types and distinct SLE features.

**Methods:**

Four hundred ninety-seven well-characterized SLE patients and 322 population controls were included. Disease activity was assessed by SLE Disease Activity Index (SLEDAI) and Systemic Lupus Activity Measure (SLAM). Functional type I IFN activity was estimated by a WISH reporter cell assay. Levels of IFN-γ were estimated by MSD 30-plex assay. IFN-α and IFN-λ1 were measured by ELISA. Values above the third quartile of patients’ measurements were defined as high. Associations among high IFN results and SLE features were investigated by nominal regression analysis.

**Results:**

All IFN measurements were higher in SLE patients than in controls. High type I IFN activity correlated with levels of IFN-γ and IFN-α and associated with active SLE in most domains: weight loss, fatigue, fever, rash, lymphadenopathy, arthritis, nephritis and haematological manifestations. Specific SLE subsets were linked to the upregulation of different subtypes of circulating IFNs: high IFN-γ to arthritis, nephritis and anti-Ro60 antibodies and high IFN-α to mucocutaneous engagement and anti-Ro52 and anti-La antibodies. Isolated high IFN-λ1 was coupled to anti-nucleosome antibodies and less severe SLE.

**Conclusions:**

High functional type I IFN activity captures active SLE in most domains, but more distinct patterns of organ involvement are associated with profiles of circulating IFNs. High IFN-γ as well as high functional type I IFN activity is a characteristic of severe SLE with nephritis and arthritis, while elevated levels of IFN-α associate with active mucocutaneous inflammation and a more benign cardiovascular profile. IFN-λ1 in isolation is associated with milder disease. Our findings suggest that IFNs contribute to the heterogeneity of clinical manifestations in SLE, and measuring circulating IFNs could assist in designing clinical trials with therapies targeting IFN pathways.

**Electronic supplementary material:**

The online version of this article (10.1186/s13075-019-1878-y) contains supplementary material, which is available to authorized users.

## Background

Systemic lupus erythematosus (SLE) is an autoimmune disease that can affect many organ systems. A common hallmark of the disease is the presence of autoantibodies against nuclear antigens (ANA). ANA may target different intranuclear molecules, including double-stranded DNA (dsDNA). It is observed that different autoantibody patterns may associate with distinct clinical manifestations. Anti-dsDNA, anti-nucleosome and anti-Sm antibodies are associated with lupus nephritis, while anti-Ro and anti-La are more common among patients with cutaneous lupus (CLE) and/or secondary Sjögren’s syndrome [1, 2].

Iatrogenic, IFN-α and IFN-γ-induced SLE cases have been reported [3, 4], and many studies thereafter have demonstrated the importance of IFNs in SLE [5, 6]. Type I IFNs include multiple subtypes of IFN-α, IFN-β, IFN**-**к, IFN-τ and IFN-ω, while IFN-γ is the only type II IFN. There are four members in the type III IFN’s (IFN-λ) family [7]. Type I and III IFNs serve in antiviral protection, and IFN-γ is a key molecule in the immune responses against a range of pathogens [7].

High serum levels of type I (IFN-α), II (IFN-γ) and III (IFN-λ1) IFNs are observed in SLE patients and have been associated with high disease activity [8–11]. Furthermore, many genetic risk factors for SLE are located along the type I IFN pathway and elevated type I IFN levels have been observed in SLE family members, supporting that enhanced type I IFN activity is a heritable risk factor [12, 13]. On the other hand, levels of IFN-γ increase years before the SLE diagnosis, while autoantibodies and increased type I IFN activity are later features, detected in the more immediate pre-disease timeframe [9]. Much of the functional type I IFN activity in SLE sera depends on IFN-αs, but also IFN-β is present and may play a role [12]. Taken together, multiple IFN types are of importance in SLE pathogenesis.

Measurements of circulating IFN-α by ELISA have been complicated by poor sensitivity and specificity [14]. IFN-α detection by dissociation-enhanced lanthanide fluoro-immunoassay (DELFIA) is more accurate, but laborious and not widely available technique [8]. Recently, a novel single-molecule array digital ELISA technique has been developed for detection of IFN-α [15]. Many earlier investigators have relied on alternative methods, such as estimating upregulation of IFN-regulated genes in patient-derived cells, the so-called IFN signature [16, 17]. Importantly, some investigators reported that type I and type II IFN signatures may overlap [18], which might be a limitation of the IFN signature method. Another commonly used method is the functional activity assay, where reporter cells are exposed to serum and the upregulation of type I IFN genes is used as a readout [2, 12]. Specificity can be ensured by blocking IFNs sequentially and by choosing cell lines that are not responsive to other IFN types [12, 19].

Given the pathological importance of several IFNs in SLE, therapeutic approaches which target IFNs are under evaluation. Clinical trials targeting type I IFN have thus far been challenging, but data supports subset effects [20–22]. These studies have blocked one type of IFN, but it seems likely that SLE patients have simultaneous upregulation of multiple IFNs, which may explain the observed incomplete responses to date. We have addressed this question in the present study via simultaneous measurement of peripheral levels of all three IFN subtypes. We have also investigated associations between IFN subtypes and various SLE manifestations, and we identified that certain organ manifestations are associated with distinct IFN subtypes.

## Patients and methods

### Study population

The study was designed in a cross-sectional manner. During the period 2004–2010, 497 patients were included at Karolinska University in Stockholm. All patients fulfilled at least four of the 1982 revised American College of Rheumatology (ACR) classification criteria for SLE [23]. Exclusion criteria were age under 18 and current pregnancy. Population controls (*n* = 322) were individually matched for age, gender and geographical region to the first 322 SLE patients. An SLE diagnosis was the only exclusion criterion for the controls. All participants underwent a structured examination by a rheumatologist. ACR SLE criteria were tabulated [23]. Disease duration was defined as the time (years) from the first time document SLE diagnosis in the patient records until inclusion into the cohort. Disease activity was assessed by the SLE Disease Activity Index (SLEDAI), a qualitative measure of active versus inactive disease in major organs, and also by Systemic Lupus Activity Measure (SLAM), a graded measure of activity in affected organs. Organ damage was assessed by Systemic Lupus International Collaborating Clinics/ACR Damage Index (SDI) [24–26]. Active organ manifestations complied with definitions of the SLAM and SLEDAI instruments, with slight modifications, as defined below [26, 27].

Total mucocutaneous activity was defined as a positive score in any of SLAM items 4–7. Only severe fatigue, limiting normal activity (SLAM score 2) was included in the analysis. In addition, renal activity was graded according to British Isles Lupus Assessment Group (BILAG) definitions: active nephritis (BILAG A-B), inactive (BILAG C-D, on dialysis or transplanted) and non-nephritis (BILAG E) [28]. There were 189 (40%) patients who had ever been diagnosed with lupus nephritis (LN), and BILAG classification was available in 154 cases. Definition of vascular event (VE) included any objectively verified arterial and/or venous event, as previously described [29].

Sera and plasma samples were collected after overnight fasting at inclusion, aliquoted and stored at − 70 °C until analysis. The study was carried out in compliance with the Declaration of Helsinki. Participants received oral and written information about the study, and all subjects provided informed written consent to participate in the study.

### Laboratory methods

Routine chemistry analyses were performed at inclusion according to standard procedures at the internationally certified laboratory at Karolinska University Hospital. ANA were analysed by indirect immunofluorescence (IFL) on Hep-2 cells (Immunoconcepts, Sacramento, CA, USA). Following autoantibody tests were performed at recruitment: ANA including sub-specificities, anti-dsDNA, anti-cardiolipin (aCL) IgG and anti-β_2_-glycoprotein1 (aβ_2_GP1) IgG antibodies, all were analysed by multiplexed bead technology (Luminex) using BioPlex 2200 system (Bio-Rad, Hercules, CA, USA). The cutoff for aCL and aβ_2_GP1 fulfilled the 99th percentile as described [30]. Lupus anticoagulant (LA) was determined by the modified Russel Viper Venom method (Biopool, Umea, Sweden) using Bioclot LA. ACL, β_2_-GP1 and LA are together referred to as antiphospholipid antibodies (aPL).

### Detection of IFNs

#### Functional assay for serum type I IFN activity

Serum type I IFN activity (IFN activity) was measured in vitro by a reporter cell assay, described elsewhere [12, 31]. In brief, WISH reporter cells (ATCC #CCL-25) were exposed to patient sera (50%) for 6 h. Afterwards, the cells were lysed, and cDNA was made from total cellular mRNA. Canonical type I IFN-induced gene expression MX1, EIF2AK2 and IFIT1 was measured by qPCR. The relative expression of three genes was standardized to healthy donor sera and summed to a score reflecting the ability of sera to upregulate IFN-induced gene expression (functional type I IFN activity) [12]. This assay does not detect type II or type III IFNs [32], and IFN-κ has not been detected in SLE sera [19].

#### Measurement of circulating IFNs

Plasma levels of IFN-γ were measured using the Mesoscale Discovery (MSD) multiplex analysis of cytokines, MSD V-PLEX™ Human Cytokine 30-plex kit (K15054D; Mesoscale Discovery, Gaithersburg, MD) according to the manufacturer’s instructions, as described before [33]. The limit of quantification was 2.5 pg/ml.

Measurements of IFN-α and IFN-λ1 were performed by commercial ELISA in 261 SLE patients and as many population controls and have been reported in our earlier study [11].

IFN-αs were measured by pan IFN-α ELISA detecting kit, and the detected IFN-α subtypes were 1/13, 2, 4, 5, 6, 7, 8, 10, 14, 16 and 17 (product code 3425-1A-20, Mabtech AB, Nacka, Sweden). Our analysis detected 12 subtypes of IFN-α (except IFN-α21), but for simplicity reasons, we will further refer to our findings as IFN-α. Mouse monoclonal anti-IFN-λ1 IgG2A capture antibody (catalogue number MAB15981, R&D Systems, Minneapolis, MN, USA) and affinity-purified goat polyclonal IgG (catalogue number BAF1598, R&D Systems) were used for coating and detection, respectively. ELISAs were performed as indicated by the manufacturer. The detection limits were set according to the manufacturer’s recommendation: at 36 pg/ml for IFN-α and 300 pg/ml for IFN-λ1.

For further analysis, patients were stratified to those with “high” levels, according to the IFN measurements, by estimating the third quartile of IFN values in the patient group.

**Fig. 1:**
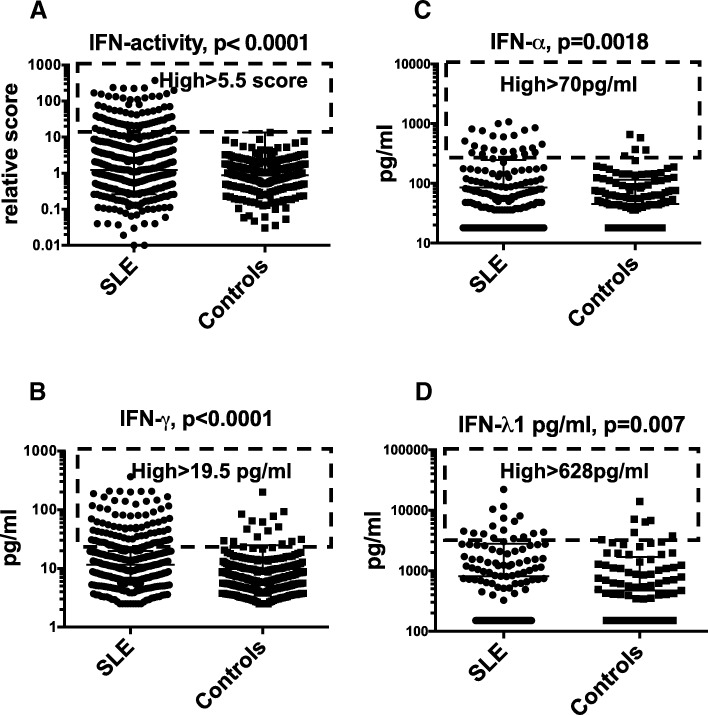
Type I IFN activity and levels of IFN-α, IFN-γ and IFN-λ1 in SLE patients and population controls. Type I IFN activity in vitro (**a**) and IFN-γ levels (**b**), IFN-α (**c**) and IFN-λ1 (**d**) were all higher in SLE patients then population controls (Mann-Whitney *U* test). The dashed boxes indicate individuals with high levels (> 75th percentile of patient measures) of each investigated IFN

The limits for “high” were defined as follows: score 5.5 for type I IFN activity, 70 pg/ml for IFN-α, 19.5 pg/ml for IFN-γ and 628 pg/ml for IFN-λ1 (Fig. 1).

### Statistical analysis

Depending on the data and the distribution, we used Student’s *t* test, Mann-Whitney or Kruskal-Wallis tests to compare continuous and ordinal data between groups. Proportions were compared by the two-tailed Fisher exact test or the Pearson chi-square test. Correlations were calculated by Spearman’s rank correlation analysis. Multivariable analysis was performed using the nominal logistic regression model and complemented with Wald test. *p* values < 0.05 were considered significant. JMP software (SAS Institute, Carey, NC, USA) was used for all statistical analyses.

## Results

### Correlation between IFN activity and IFN-γ levels

Basic characteristics of the cohort are presented in Table 1. Functional type I IFN activity and IFN-γ levels were higher in SLE than in controls (Table 1 and Fig. 1).

**Table 1:** Characteristics of the cohort

Characteristics	SLE, *n* = 497	Population controls*, n* = 322	*p* value
Age, *M* (SD)	46 (15)	47.8 (14.7)	ns
Gender (male/female)	67/429	26/296	ns
Caucasians	89%	97%	< 0.0001
Current smoking	18.5%	14%	ns
Malar rash	48.5%	–	
Discoid rash	18%	–	
Photosensitivity	63%	–	
Oral ulceration	34%	–	
Arthritis	82%	–	
Serositis	40%	–	
Nephritis	42%	–	
Neuropsychiatric (NPSLE)	11.5%	–	
Leukopenia	48%	–	
Lymphopenia	54%	–	
Thrombocytopenia	20%	–	
Haemolytic anaemia	6%	–	
Positive ANA, ever	99%	nd	
Positive anti-dsDNA, ever	67%	nd	
SLAM > 6	49%	–	
SLEDAI > 6	26%	–	
SDI > 0	64%	–	
Arterial events	11%	1.25%	< 0.0001
Venous thromboembolic events	16.5%	1.25%	< 0.0001
Any vascular events	24%	2.5%	< 0.0001
Prednisolone dose^^^, M (SD)	9 (45) mg	na	
Prednisolone 10 mg or more	25%	na	
Mean and standard deviation of the measurements			
Type I IFN activity	12.1 (36)	1.3 (1.5)	< 0.0001
IFN-α pg/ml	161.4 (161)	45.1 (69)	0.0002
IFN-γ pg/ml	25.9 (79)	13.5(69)	0.02
IFN-λ1 pg/ml	811.2 (1989)	472.3 (1208)	0.01
Proportions of the groups with high IFN levels			
Type I IFN activity^H^ (score > 5.5)	25%	2%	< 0.0001
	25%	6.5%	< 0.0001
IFN-α^H^	25%	14.5%	0.003
IFN-γ^H^ (> 19.5 pg/ml)	25%	6.5%	< 0.0001
IFN-λ1^H^	25%	13.5%	0.0009

Characteristics of SLE, as defined by 1982 ACR SLE classification criteria, if ever observed [23]. Student *t* test and Mann-Whitney tests were used for comparison

*SLAM*, systemic lupus activity measure; *SLEDAI*, SLE Disease Activity Index; *SDI*, SLE disease damage index; *arterial events*, objectively verified coronary heart disease or stroke; *venous thromboembolic events*, pulmonary or/and deep venous thrombosis, any vascular events include history of either arterial or venous events, or both; *nd*, not done, *na*, not applicable

^H^Patients with IFN levels over the third quartile were defined as high expressers

^^^Prednisolone dose or bioequivalent steroid dose

IFN activity correlated with serum concentrations of IFN-γ and IFN-α and weakly with IFN-λ1 (Table 2 and Additional file 1: Figure S1).

**Table 2:** Correlation between serum IFN activity; levels of IFN-α, IFN-γ and IFN-λ1; disease activity and damage

Parameter	IFN activity (*ρ*)	*p* value	IFN-γ (*ρ*)	*p* value
IFN-α	0.25	< 0.0001	0.2	0.009
IFN-γ	0.4	< 0.0001	–	–
IFN-λ1	0.12	0.04	0.05	ns
SLAM	0.3	< 0.0001	0.08	ns
SLEDAI	0.3	< 0.0001	0.14	0.005
SDI	− 0.13	0.003	− 0.02	ns
Prednisone dose	0.07	ns	− 0.06	ns

Statistical analysis was performed by Spearman rank correlation test. Results are presented as Spearman rank correlation coefficient (*ρ*)

*SLAM* Systemic Lupus Activity Measure, *SLEDAI* SLE Disease Activity Index, *SDI* SLE disease damage index

### Patients with high levels of different IFN types have different SLE features

We hypothesized that high levels of the different IFNs could have different manifestations of active SLE. We thus identified patients with the highest levels of each measurement (over the third quartile) and grouped accordingly: those with high type I IFN activity or high IFN-γ (Fig. 1). Data on IFN-α and IFN-λ1 has been published before, but is included in Additional file 2: Tables S1 and S2 to allow comparison. In the statistical analysis, each group was compared to the rest of the patients.

High type I IFN activity and high IFN-γ associated with active SLE (SLEDAI > 6 and SLAM > 6) and correlated positively with disease activity scores (Table 2 and Additional file 2: Table S1). High type I IFN activity was associated with younger age, shorter disease duration and less disease damage (Additional file 2: Table S1).

Constitutional symptoms, including weight loss, severe fatigue and fever, were also associated with high type I IFN activity. Lymphadenopathy, arthritis and active lupus nephritis (LN) were all more common among those with either high type I IFN activity or high IFN-γ measurement (Additional file 2: Table S1).

Overall mucocutaneous involvement (SLAM items 4–7) was associated with type I IFN activity and high levels of circulating IFN-α. Even separate parameters such as new rash, mucosal-acute cutaneous LE (ACLE), discoid LE (DLE) and alopecia (Additional file 2: Table S1), all were more common among those with high functional type I IFN activity.

Severe neuropsychiatric SLE (NPSLE, as defined seizures or psychosis (ACR 1982 criteria [23])) was somewhat less common among those with high type I IFN activity (Additional file 2: Table S1).

### Patients with high levels of different IFN types have different autoantibody profiles and laboratory features

Haematological manifestations, including anaemia, leukopenia, lymphopenia, thrombocytopenia and high erythrocyte sedimentation rate (ESR), all associated with high type I IFN activity, as well as with high IFN-γ. Low complement was linked to high type I IFN activity and high levels of circulating IFN-α and IFN-γ (Additional file 2: Table S2).

High type I IFN activity associated with the classical SLE autoantibodies against dsDNA, nucleosomes, Sm, SmRNP, RNP68, Ro52, Ro60 and La. All, except anti-nucleosomes and anti-La, were also more common among the IFN-γ high group. High IFN-α associated positively with anti-Ro52, anti-Ro60 and anti-La autoantibodies, but negatively with aPL specificities (Additional file 2: Table S2), while only anti-nucleosome antibodies were more common among IFN-λ1 high’s.

There were no associations between aPL, secondary APS or history of vascular events (VE) neither with type I IFN activity nor with IFN-γ levels, though fewer patients were on warfarin treatment in the IFN-γ high group. History of vascular events was less common in the IFN-α high group. Interestingly, the frequency of vascular events, LA, triple positivity for aPL and warfarin prescription were numerically more common among those with high IFN-λ1, but did not reach statistical significance (Fig. 3). Raynaud’s phenomenon associated positively with high IFN activity (Additional file 2: Tables S1 and S2).

### Multivariable analysis demonstrates that high IFN levels associate with different SLE features

Next, we performed multivariable analysis on four patient groups, grouped by high levels of each IFN measurement as a covariate in order to identify what SLE features are associated with high levels of circulating IFNs. In our cohort, there were altogether 248 SLE cases in whom all four IFN measurements were performed.

We hypothesized that high levels of different IFN subtypes may associate with distinct patterns of organ involvement. In Fig. 3a–d, we demonstrate how IFN measurements distribute among patients with different SLE manifestations, and stratified nominal regression analysis with statistical results is presented in Table 3.

**Table 3:**
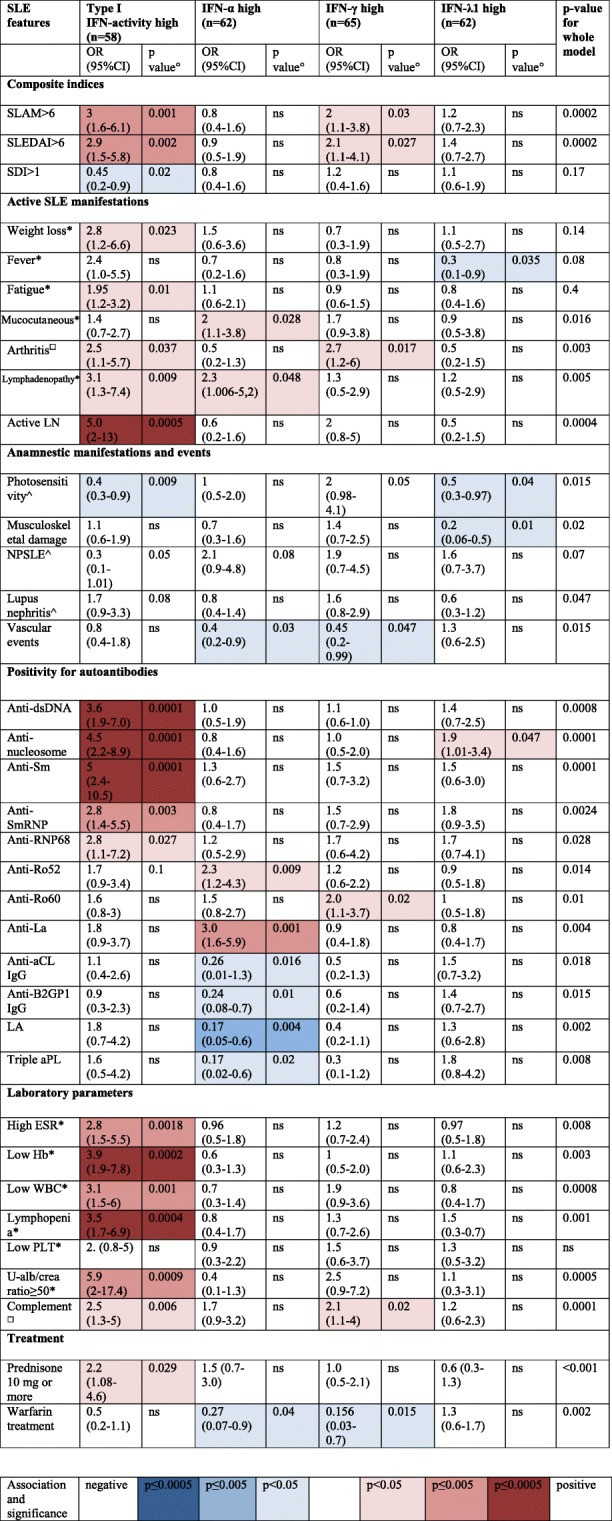
The associations among high IFN levels and SLE manifestations (stratified analyses of nominal regression models)

The analysis was run on 248 patients, in whom all four IFN measurements were available

*NPSLE* neuropsychiatric SLE, *LN* lupus nephritis, *ESR* erythrocyte sedimentation rate, *Hb* haemoglobin, *WBC* white blood cells, *PLT* platelets, *AVE* any vascular event, *aCL* anti-cardiolipin, *B2GP1* beta 2 glycoprotein 1, *LA* lupus anticoagulants, *aPL* antiphospholipid abs, *U-alb/krea* urine albumin kreatinine ratio

*Definition by Systemic Lupus Activity Measure (SLAM)

^□^Definition by SLE Disease Activity Index (SLEDAI)

^^^Definition by 1982 ACR SLE classification criteria, if ever observed, disease damage was defined by SLE disease damage index (SDI)

^°^*p* values based on Wald test

Stratified analyses of nominal logistic regression models confirmed that high functional type I IFN activity was positively associated with active disease, including arthritis, nephritis and lymphadenopathy. The associated serologic parameters included positivity for anti-dsDNA, anti-nucleosome, anti-Sm, anti-SmRNP and anti-RNP68 autoantibodies; low complement; high ESR and majority haematological manifestations, excluding thrombocytopenia. Weak negative associations were found for photosensitivity (Table 3 and Fig. 3).

High levels of IFN-γ were associated with high disease activity, and clinical features such as active arthritis, anti-Ro60 and low complement, as well as active nephritis (Table 3, Fig. 3 and Additional file 2: Table S1). Prescription of warfarin had a weak negative association, as well as lower OR for past vascular events.

Elevated IFN-α levels were coupled to active mucocutaneous disease, anti-Ro52 and anti-La. Further, we observed that the IFN-α high group had lower OR for the presence of aPLs, history of vascular events and warfarin treatment (Table 3 and Fig. 3).

High IFN-λ1 associated positively with anti-nucleosome antibodies, while ORs for fever, photosensitivity and musculoskeletal damage were lower in this subset (Table 3). A numerical, but non-significant association with aPL autoantibodies was observed (Fig. 3).

### Clinically relevant overlaps among upregulated IFNs

**Fig. 2:**
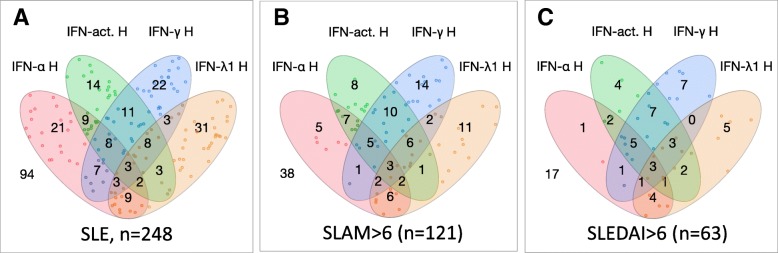
The distribution of the proportions of patients with high levels of each IFN measurement. The Venn diagram depicts how patient groups with high levels of different IFNs distribute and overlap in the cohort (**a**), and among those with active SLE as scored by SLAM (**b**) and SLEDAI (**c**). Only patients in whom all four measurements were available are included in the analysis (*n* = 248). The number outside diagram indicates the number of patients who had none of the IFNs expressed at a high level (> 75th percentile of patient measures)

**Fig. 3:**
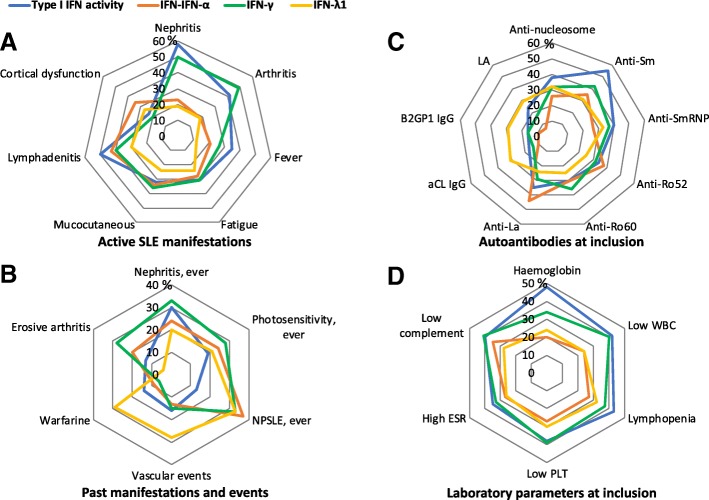
Distribution of IFN-high subsets among SLE patients with different characteristics. The figure illustrates how subsets of different IFN types distribute among patients with active SLE manifestations (**a**), past manifestations and events (**b**), positivity for autoantibodies (**c**) and laboratory parameters (**d**) (presented in %) as assessed at inclusion. Abbreviations: LN lupus nephritis, NPSLE neuropsychiatric SLE (*at inclusion, NPSLE was classified by 1982 ACR criteria, seizures or psychosis), aCL anti-cardiolipin, B2GP1 β2glycoprotein-I, LA lupus anticoagulant, ESR erythrocyte sedimentation rate, WBC white blood cells, PLT platelets. Only patients in whom all four measurements were available are included in the analysis (*n* = 248)

High levels of several IFN types were observed among subsets of patients with different characteristics, indicating that a variety of IFNs could have roles (Figs. 2 and 3). A proportion of patients with active SLE (12% with SLAM > 6 and 28.5% with SLEDAI > 6) had simultaneously high type I IFN activity and high IFN-γ (Fig. 2).

We sorted out patients displaying active disease by each particular manifestation and investigated proportions of the upregulated IFNs. Data is presented in Venn diagrams (Additional file 1: Figure S2). Only patients in whom all four IFN measurements were available were included in the analysis (*n* = 248). There were 30 patients who had simultaneously high IFN activity and high IFN-γ; they comprised 38.5% of those with active nephritis (Fig. 3 and Additional file 1: Figure S2). A separate nominal regression analysis was run on three subgroups: double IFN activity and IFN-γ high, IFN-α and IFN-λ1 highs three co-variates. The double high group had higher OR for active nephritis (7.4, CI 2.8–19.2). Besides, the overlap between high IFN activity and IFN-γ was observed among patients with arthritis (27%) and low complement (25%) and carrying anti-dsDNA (16%), anti-Sm (33%) or anti-Ro60 (17.5%) antibodies (Additional file 1: Figure S2).

## Discussion

Here, we present the first comparative study on functional type I IFN activity measured in vitro by WISH reporter cell assay, a method often used as a golden standard in estimating IFN signature in SLE, and measurements of circulating type I, type II and type III IFNs. Our study is unique as we measured all three IFN types in a large and very well-characterized SLE cohort. This approach allowed us to determine how different IFN subtypes associate with each other and with clinical SLE subsets.

We found that type I IFN activity correlates with circulating levels of type I and type II IFNs, but only weakly with type III IFN, which to our knowledge has not been observed before. It has been known that levels of IFN-γ increase years before the SLE diagnosis. The observed correlation between IFN activity and IFN-γ implicates that the role of IFN-γ might be as important as type I IFNs in SLE. Consequently, IFN-γ could also be an important target for therapeutic strategies. Further, multivariable nominal regression models allowed us to identify phenotypic SLE subsets associated with high levels of different IFN subtypes.

We report a novel observation that active LN associates with both high IFN activity, but also high levels of circulating IFN-γ. Among cases with lupus nephritis (either active or past), we observed a proportion of patients with overlapping elevation of IFN activity, IFN-γ and serum IFN-α, as reported before [10, 32]. Thus, our findings extend previous knowledge that renal flares associate with a combined IFN-α and IFN-γ gene signature [32, 33]. Besides IFN-γ, other IFN types seem to be of importance. A study from the Asian population reported that also high IFN-λ1 levels are associated with LN [34]. We could not confirm this association, possibly due to the fact that some patients were already on induction therapy at recruitment; nevertheless, in our earlier study, we found that high levels of IFN-λ1 were linked to poor renal outcomes [11]. Interestingly, in our cohort, high serum IFN-α levels associated with better preserved renal function, and this observation remained after age adjustment [11].

SLE arthritis was linked to both high type I IFN activity and high IFN-γ. Previously, we reported that arthritis was associated with the upregulation of IP-10/CXCL-10 [11]. Altogether, our observations suggest that arthritis in SLE is associated with several cytokines within the IFN pathways [11].

In line with other investigators, our data demonstrate that mucocutaneous involvement associates with high type I IFN activity in vitro and with high levels of circulating IFN-α [8, 35], suggesting that this subgroup might benefit from IFN-α blocking agents [20].

We also observed that autoantibody patterns are coupled to different IFN subtypes.

Type I IFN activity aligned with the majority of ANA sub-specificities as reported before [8, 31, 32, 35]. In multivariable analysis, we identified a novel association that high IFN-γ is most strongly linked to anti-Ro60. We confirm previous data on IFN-α, known to be coupled to anti-Ro52 and anti-La [8].

Few studies have investigated the relationship between IFNs and vascular outcomes in SLE [36]. Earlier reports from broader ethnic populations observed an association of aPL and high type I IFN activity in certain ancestral groups [37]. In the current, mainly Caucasian cohort, multivariable analysis demonstrated lower likelihoods for vascular events, aPL and warfarin prescription for those with isolated high IFN-α. Also, the IFN-γ high group seemed to have slightly lower frequency of vascular events, as prescription of anticoagulant treatment was less common. High IFN-λ1 was linked to anti-nucleosome autoantibodies, and numerically, but not significantly, with higher frequencies of aPL, vascular events and warfarin use. Our findings suggest that in this Caucasian population, type I and type II IFNs are primarily coupled to autoreactivity to intracellular auto-antigens, but not to aPLs, that target non-nuclear structures such as plasma proteins and membrane phospholipids [30]. To our knowledge, associations between cardiovascular morbidity and levels of peripheral levels of IFNs type II and III have not been reported before.

It has been known that high type I IFN activity is associated to haematological manifestations [35]. Our report adds novel information that haematological manifestations are also associated with elevated IFN-γ. As expected, we confirm that low complement levels follow high type I IFN activity, and also high IFN-α and IFN-γ levels [2, 9, 28, 29].

A substantial proportion of earlier investigators relied on estimations of IFN-gene upregulation and their associations with SLE disease activity [32, 37, 38]. However, every investigator chose to measure different IFN-regulated genes, and therefore, studies are difficult to compare. Another complicating factor in the estimation of IFN signature is that the same genes can be regulated by several IFN types. Our findings demonstrate that active SLE patients have high levels of circulating type I, type II and type III IFNs and that different organ involvement seems to be coupled to different IFN types. The presented data suggest that measuring circulating IFN levels is more informative that estimating IFN signature and could have a role for identifying patients who will benefit from anti-IFN therapies in SLE.

We demonstrate that several major clinical manifestations associate with a dominating IFN type, but still proportions of patients also have other upregulated IFN types. It would be of interest if our observations could be confirmed in other cohorts. In clinical practise, it would be of importance to measure all three IFN types and identify a dominating one in order to tailor directed therapy. Further, our findings indicate that compounds blocking several IFN pathways within the cell would be a more promising therapeutic approach rather than monoclonal antibodies blocking a single circulating IFN or its receptor [20, 39].

Activation of type I IFN-regulated pathways is thought to be a key mechanism in SLE pathogenesis [40], but emerging evidence demonstrate that also IFN type II and the Th17 cytokine axis have important roles [9, 11, 41]. A phase 2 trial on ustekinumab, antibodies blocking IL-12/IL-23 pathway, in SLE was recently published with positive and promising results [42]. Good response to ustekinumab was associated with declining IFN-γ levels, while type I IFN signature scores did not decline. We previously reported that high levels of IL-23 and IL-17 are associated to treatment-resistant LN with poor outcomes [10, 36, 37]. Altogether, accumulating evidence supports a role for IFN-γ and the Th17 axis in SLE, suggesting that severe manifestations, including LN, could benefit from IFN-γ- and/or Th17-blocking agents.

IFN-β is an important member of the type I IFN family. Unfortunately, we did not measure circulating levels of IFN-β. It is possible that some of the discordance in our results between the functional type I IFN assay and the IFN-α ELISA are due to the fact that the functional assay also measures IFN-β. While this could be an explanation, it has been shown that IFN-α is the major circulating type I IFN in most SLE sera [12]. Thus, it seems likely that the functional assay is more sensitive than the ELISA for overall IFN-α measurement [14].

We observed that patients with high IFN activity had slightly higher ORs for prednisolone doses over 10 mg, but did not observe any other associations between IFN levels and immunomodulating therapies. The design of the presented study was cross-sectional, and patients with highly variable disease activity scores as well as different treatments were included. This fact could have limited our possibility to analyse how different treatments could influence IFN levels. A prospective longitudinal study would be a better option to study these questions.

## Conclusions

We demonstrate that high functional type I IFN activity in vitro correlates with circulating levels of IFN-γ, as well as IFN-α. We confirm that high type I IFN activity associate with active SLE in the majority domains. Further, high levels of IFN-γ associate with nephritis and arthritis, while high levels of IFN-α associate with mucocutaneous disease, thus suggesting that specific organ involvements associate with different IFN subtypes. These observations are of major importance for understanding disease mechanisms and for individual tailoring of IFN-targeting therapies in SLE.

## Additional files

Additional file 1: **Figure S1.** Scatter plots of correlations among type I IFN activity and IFN-α (A), IFN-γ (B) and IFN-λ1 (C). **Figure S2.** Clinically relevant overlaps among upregulated IFNs. Each Venn diagram depicts patient group with a certain SLE manifestation (as defined in the “Methods” section) and what numbers of patients within the group had high measurement of each IFN. Only patients in whom all four measurements were available were included in the analysis (*n* = 248). On the left side of Venn diagrams, the number indicates in how many patients none of the IFNs were expressed at high level (> 75th percentile of patient measures). N- indicates how many patients out of 248 had the certain manifestation. Abbreviations: H high, SDI SLE disease damage index, NPSLE neuropsychiatric SLE, *classified according to 1982 ACR criteria seizures and/or psychosis, APL antiphospholipid antibodies. (PDF 1436 kb)

Additional file 2: **Table S1.** High IFN activity and high levels of IFN-α, IFN-γ and IFN-λ1 associate with different clinical manifestations of active SLE. **Table S2.** High IFN activity and high levels of IFN-α, IFN-γ and IFN-λ1 associate with different serologic and laboratory findings, and steroid and warfarin prescription. (DOCX 44 kb)
