# Supplementary figures and images for: High levels of circulating interferons type I, type II and type III associate with distinct clinical features of active systemic lupus erythematosus
Source: Arthritis Res Ther. 2019 Apr 29;21:107. doi: 10.1186/s13075-019-1878-y (PMC6489203; doi:10.1186/s13075-019-1878-y)

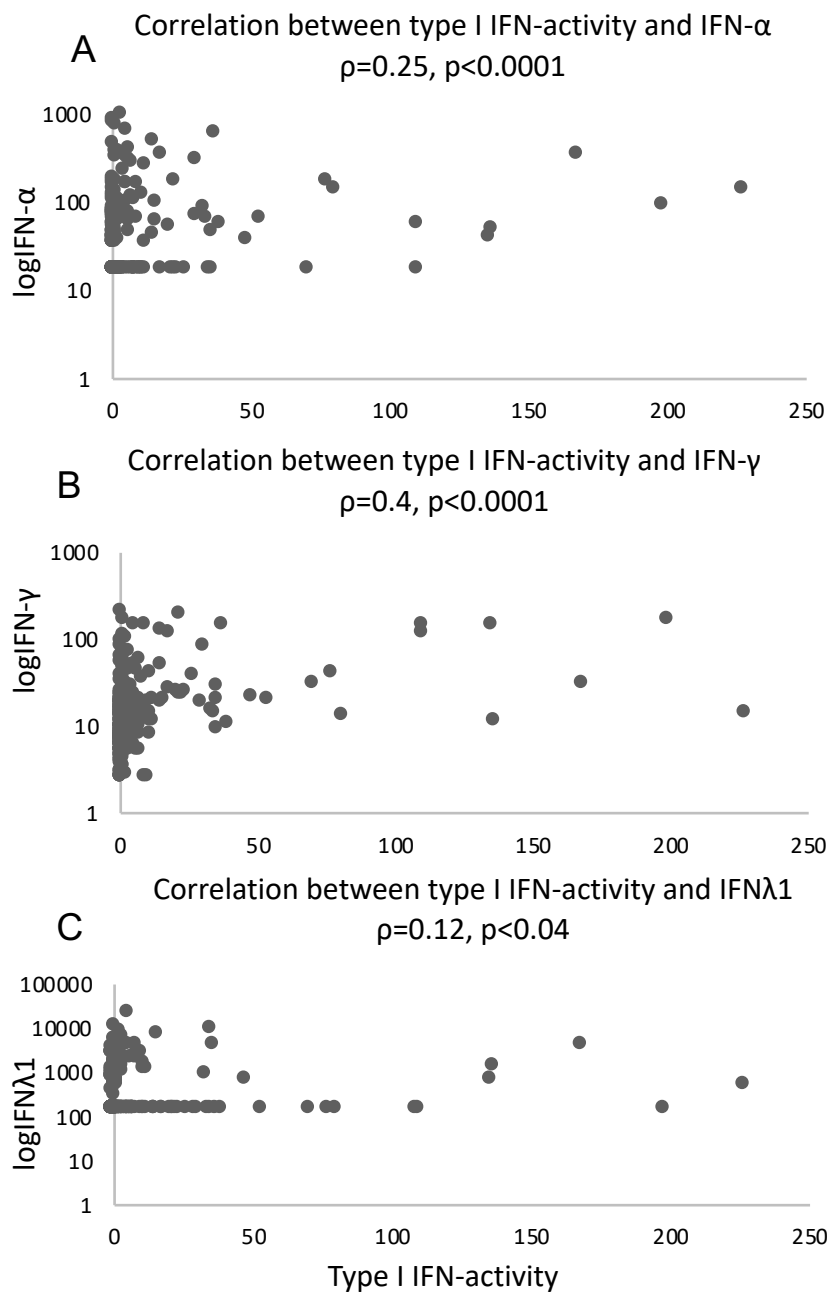

Active manifestations

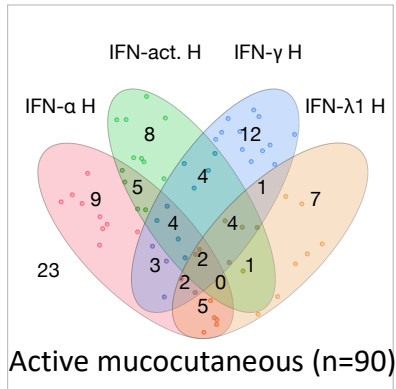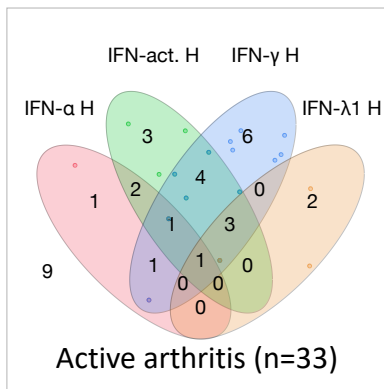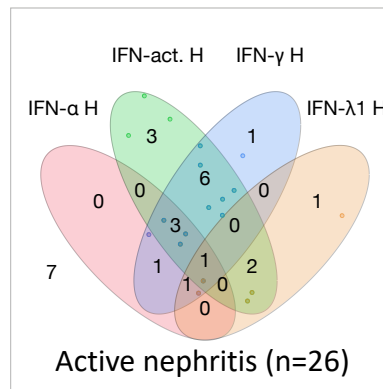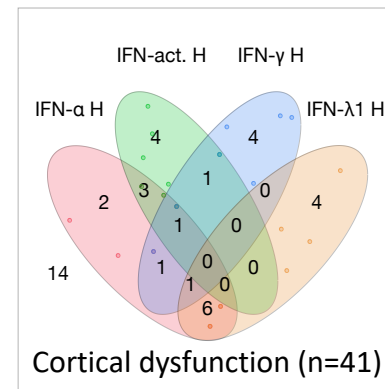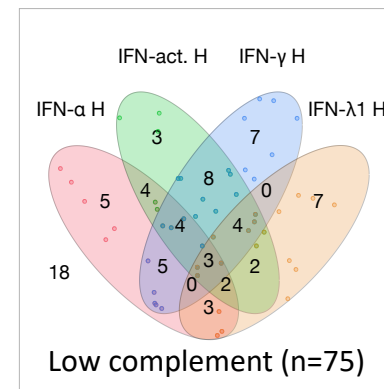

Autoantibodies

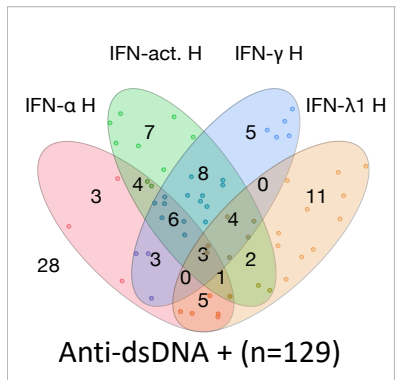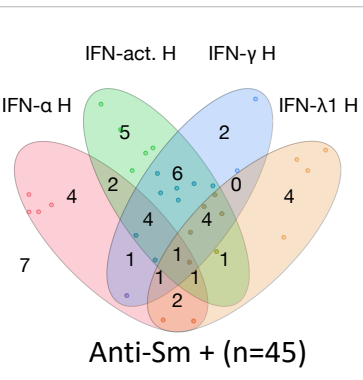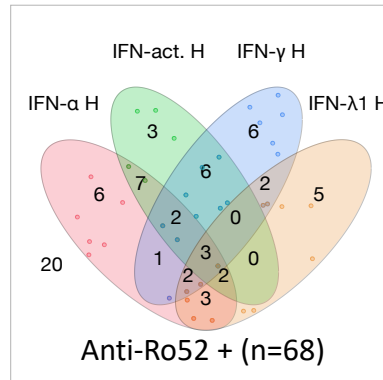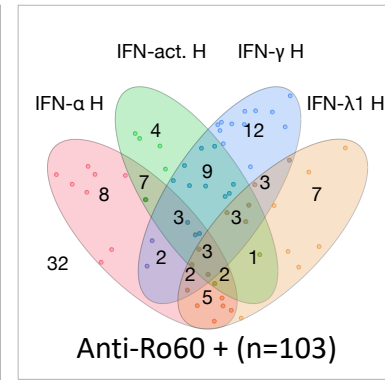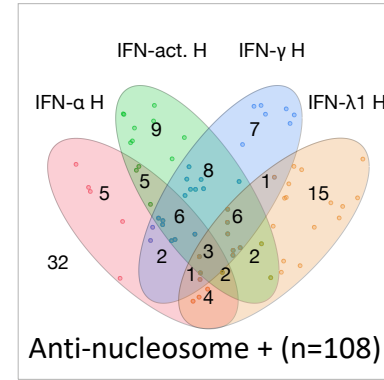

Past manifestations and events

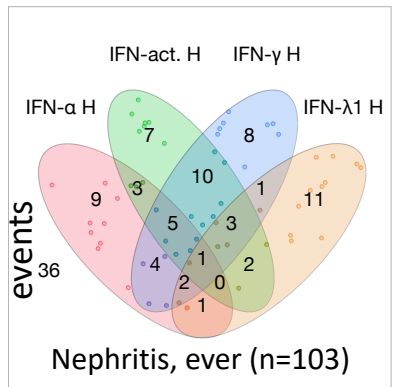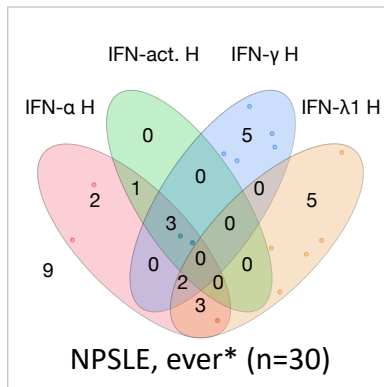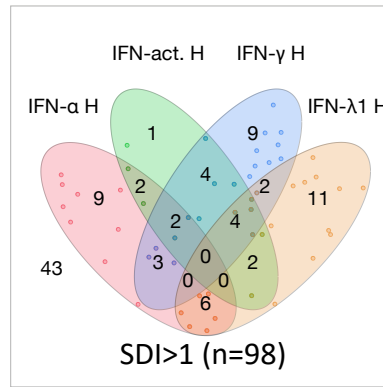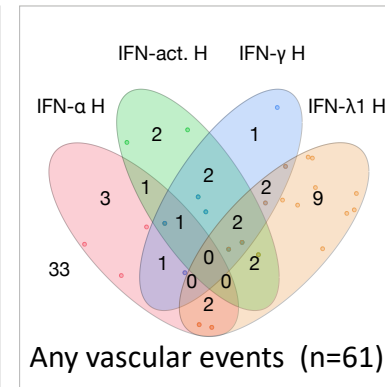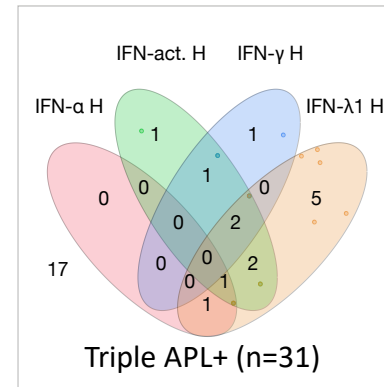

Supplement: Supplementary file 1 — Figure S1. Scatter plots of correlations among type I IFN activity and IFN-α (A), IFN-γ (B) and IFN-λ1 (C). Figure S2. Clinically relevant overlaps among upregulated IFNs. Each Venn diagram depicts patient group with a certain SLE manifestation (as defined in the “Methods” section) and what numbers of patients within the group had high measurement of each IFN. Only patients in whom all four measurements were available were included in the analysis (n = 248). On the left side of Venn diagrams, the number indicates in how many patients none of the IFNs were expressed at high level (> 75th percentile of patient measures). N- indicates how many patients out of 248 had the certain manifestation. Abbreviations: H high, SDI SLE disease damage index, NPSLE neuropsychiatric SLE, *classified according to 1982 ACR criteria seizures and/or psychosis, APL antiphospholipid antibodies. (PDF 1436 kb) [file 13075_2019_1878_MOESM1_ESM.pdf]
