# Supplementary material for: High levels of circulating interferons type I, type II and type III associate with distinct clinical features of active systemic lupus erythematosus
Source: Arthritis Res Ther. 2019 Apr 29;21:107. doi: 10.1186/s13075-019-1878-y (PMC6489203; doi:10.1186/s13075-019-1878-y)
Supplement: Supplementary file 2 — Table S1. High IFN activity and high levels of IFN-α, IFN-γ and IFN-λ1 associate with different clinical manifestations of active SLE. Table S2. High IFN activity and high levels of IFN-α, IFN-γ and IFN-λ1 associate with different serologic and laboratory findings, and steroid and warfarin prescription. (DOCX 44 kb) [file 13075_2019_1878_MOESM2_ESM.docx]

**Table S1. High IFN-activity and high levels of IFN-α, IFN-γ and IFN- λ1 associate with different clinical manifestations of active SLE**

| Parameter | All SLE n=497 | IFN-activity^H^  n=119 | Other  n=378 | P-value | IFN-α^H^  n=65 | Other  n=194 | P-value | IFN-γ^H^  n=113 | Other  n=384 | P-value | IFN- λ1^H^ n=65 | | Other  n=194 | p-value |
| --- | --- | --- | --- | --- | --- | --- | --- | --- | --- | --- | --- | --- | --- | --- |
| **Demographic data M (SD)** | | | | | | | | | | | | | | |
| Age at inclusion, y | 47.6 (14) | 40 (14) | 48 (15) | 1x10^-4^ | 43 (15) | 49 (14) | 0.005 | 45 (16) | 46 (14) | ns | 46 (15) | | 48 (14) | ns |
| Disease duration, y | 15 (11) | 9 (11) | 13 (11) | 2 x10^-4^ | 12.8 (11) | 16 (11) | 0.02 | 14 (12) | 12 (11) | ns | 30 (13) | | 33 (14) | ns |
| **Composite indices (%)** | | | | | | | | | | | | | | |
| SLAM>6 | 50 | 70.5 | 43 | <1x10^-4^ | 49 | 49 | ns | 56 | 44 | 0.04 | 52 | | 48 | ns |
| SLEDAI>6 | 27 | 45.5 | 21 | <1x10^-4^ | 29 | 25 | ns | 36 | 22 | 0.006 | 31 | | 25.5 | ns |
| SDI>1 | 37.5 | 24.5 | 41 | 5x10^-4^ | 34.5 | 42 | ns | 34.5 | 38 | ns | 41.5 | | 40 | ns |
| **Active SLE manifestations (%)** | | | | | | | | | | | |  |  |  |
| Weight loss* | 11 | 18 | 8.5 | 0.009 | 15.5 | 10 | ns | 9 | 11 | ns | 12 | | 11 | ns |
| Fatigue* | 34.5 | 45.5 | 31 | 0.005 | 31 | 27.5 | ns | 32.5 | 32 | ns | 26 | | 29 | ns |
| Fever* | 13 | 20 | 11 | 0.01 | 11 | 13 | ns | 14 | 13 | ns | 6 | | 15 | 0.06 |
| Mucocuta-neous, any* | 40.5 | 50.5 | 37 | 0.01 | 52.5 | 32 | 0.005 | 44 | 37 | ns | 37 | | 37 | ns |
| Mucosal, ACLE* | 16.5 | 25.5 | 13.7 | 0.006 | 19.5 | 11 | ns | 19 | 15 | ns | 17 | | 11.5 | ns |
| Discoid CLE* | 15 | 21 | 13 | 0.05 | 20 | 15 | ns | 19 | 13.5 | ns | 14 | | 17 | ns |
| Alopecia^☐^ | 24 | 32 | 21.5 | 0.03 | 27 | 19 | ns | 25.4 | 18 | ns | 9 | | 11 | ns |
| Arthritis^☐^ | 15.5 | 42 | 20.4 | 0.01 | 18 | 26 | ns | 48.5 | 23 | 0.005 | 9 | | 14 | ns |
| Lymphadenopathy* | 13 | 22 | 10 | 0.002 | 22 | 10 | 0.02 | 19 | 10 | 0.04 | 15 | | 12 | ns |
| Active lupus nephritis | 9.5 | 58 | 19.5 | 1x10^-4^ | 11 | 11 | ns | 50 | 23 | 0.008 | 9 | | 12 | ns |
| Cortical  dysfunction* | 17 | 16.5 | 17 | ns | 21.5 | 15 | ns | 12 | 17.5 | ns | 17 | | 16.5 | ns |
| Serositis* | 8 | 14 | 6 | 0.009 | 11 | 5 | 0.07 | 8 | 7.5 | ns | 8 | | 6 | ns |
| Raynaud* | 25 | 35 | 22 | 0.006 | 28 | 22.5 | ns | 31 | 22 | 0.05 | 28 | | 22.5 | ns |
| **Anamnestic manifestations and events (%)** | | | | | | | | | | | | | | |
| Photosensitivity^ | 63.5 | 54.5 | 67 | 0.02 | 68 | 70.5 | ns | 64.5 | 64.5 | ns | 60 | | 73 | 0.06 |
| Musculosceletal damage | 15 | 16 | 14.5 | ns | 19 | 13.5 | ns | 14 | 17 | ns | 4.5 | | 20.5 | 0.002 |
| NPSLE^ | 11 | 6 | 13 | 0.04 | 13 | 9 | ns | 17 | 11 | ns | 15.5 | | 11 | ns |
| NPSLE damage | 22 | 12.6 | 25 | 0.004 | 23 | 21.5 | ns | 19.5 | 21 | ns | 26 | | 20.5 | ns |
| Lupus nephritis^ | 40 | 48 | 37.5 | 0.05 | 40 | 43 | ns | 54 | 35 | 0.0007 | 34 | | 44 | ns |
| Vascular events | 23 | 18 | 25 | ns | 12 | 29 | 0.007 | 17 | 26.5 | 0.05 | 27.5 | | 24 | ns |

| Proportions and significance | lower | p≤0.0005 | p≤0.005 | p<0.05 |  | p<0.05 | p≤0.005 | p≤0.0005 | higher |
| --- | --- | --- | --- | --- | --- | --- | --- | --- | --- |

The left column (all SLE) demonstrates distribution of investigated parameters for the whole SLE cohort. In the statistical analyses the subgroup of patients with high (above 3^rd^ quartile, abbreviated as ^H^) cytokine levels was compared to all the other patients. Statistical analyses of continuous variables were performed by Student t-test; values calculated as ratios were compared by non-parametric Mann-Whitney or Wilcoxon/Kruskal-Wallis test; all proportions were compared by 2 tailed Fishers exact test; and only for serositis (pericarditis or pleuritis) Pearson Chi-square test was applied, due to few cases. *****- indicates definition by SLAM; **^☐^**- indicates definition by SLEDAI, (>0). Any mucocutaneous activity was defined as presence of any of SLAM items from 4 to 7; for severe fatigue, SLAM definition >1 was applied. ^- indicate disease manifestations according to ACR 1982 criteria were recorded if ever present. Abbreviations: p – p-value, ACLE - acute cutaneous lupus erythematosus, NPSLE -neuropsychiatric SLE; disease damage was assessed by SLE damage index (SDI), Ns – not significant, NR – not relevant. Number and percentages between the groups differ, since all IFN measurements were not available in all the subjects due to technical reasons.

**Table S2. High IFN-activity and high levels of IFN-α, IFN-γ and IFN- λ1 associate with different serologic and laboratory findings, and steroid and warfarin prescription**

| Parameter | All SLE n=497 | IFN-activity^H^  n=119 | Other  n=378 | P-value | IFN-α^H^  n=65 | Other  n=194 | P-value | IFN-γ^H^  n=113 | Other n=384 | P-value | IFN-λ1^H^ n=65 | Other n=194 | P-value |
| --- | --- | --- | --- | --- | --- | --- | --- | --- | --- | --- | --- | --- | --- |
| **Positivity for autoantibodies (%)** | | | | | | | | | | | | | |
| Anti-dsDNA | 39 | 60.5 | 31.5 | <1x10^-4^ | 40 | 35 | ns | 44 | 35 | ns | 41.5 | 35 | ns |
| Anti-Nucleosome | 46 | 71.5 | 34 | <1x10^-4^ | 46 | 43 | ns | 51 | 41 | 0.07 | 55.5 | 40 | 0.03 |
| Anti-Sm | 18.5 | 42 | 11 | <1x10^-4^ | 24.5 | 17 | ns | 28.5 | 14 | 0.001 | 23 | 17.5 | ns |
| Anti-SmRNP | 27 | 49 | 20 | <1x10^-4^ | 26 | 25.5 | ns | 33.5 | 21 | 0.01 | 34 | 23 | ns |
| Anti-RNP68 | 10.5 | 24.5 | 6 | <1x10^-4^ | 12 | 9 | ns | 17 | 7.5 | 0.009 | 14 | 8 | ns |
| Anti-Ro52 | 28.5 | 39.5 | 25 | 0.003 | 41.5 | 22.5 | 0.004 | 37 | 25.5 | 0.02 | 27.5 | 27 | ns |
| Anti-Ro60 | 42 | 56 | 37.5 | 6x10^-4^ | 51 | 37 | 0.06 | 52 | 38 | 0.01 | 41.5 | 40 | ns |
| Anti-La | 23 | 30 | 20 | 0.03 | 37 | 16.5 | <1x  80^-5^ | 29 | 20.5 | 0.06 | 20 | 22 | ns |
| aCL IgG | 19 | 12.5 | 21 | ns | 9.5 | 22 | 0.02 | 15 | 21 | ns | 24.5 | 17 | ns |
| B2GP1 IgG | 20 | 12.5 | 22 | 0.048 | 9.5 | 24 | 0.01 | 16 | 21.5 | ns | 24.5 | 19 | ns |
| LA | 18 | 13.5 | 19 | ns | 6 | 23 | 0.002 | 13.5 | 19 | ns | 21.5 | 18 | ns |
| Tripple aPL | 11 | 7 | 12 | ns | 4.5 | 16 | 0.02 | 8.5 | 12 | ns | 18.5 | 11.5 | ns |
| **Laboratory parameters (%)** | | | | | | | | | | | | | |
| High ESR* | 41.5 | 62 | 34 | <1x10^-4^ | 43 | 42 | ns | 53 | 34 | 0.002 | 40.5 | 42.5 | ns |
| Low Hb* | 27 | 48 | 20 | <1x10^-4^ | 20 | 25 | ns | 34 | 23 | 0.02 | 24.5 | 23 | ns |
| Low WBC* | 21 | 34 | 17 | 2x10^-4^ | 24.5 | 25 | ns | 29 | 19.5 | 0.04 | 24.5 | 25 | ns |
| Lymphopenia* | 2 | 40 | 19 | <1x10^-4^ | 26 | 24.5 | ns | 32.5 | 21 | 0.02 | 31 | 23 | ns |
| Low PLT* | 10.5 | 21 | 7 | 1x10^-4^ | 12 | 11 | ns | 16 | 9 | 0.06 | 14 | 10 | ns |
| U-alb/crea ratio≥50 | 11 | 20 | 7.5 | 7x10^-4^ | 8.5 | 10.5 | ns | 16 | 7.5 | 0.013 | 12 | 9 | ns |
| Complement^☐^ | 30.5 | 49 | 24 | <1x10^-4^ | 41.5 | 27.5 | 0.04 | 39 | 26 | 0.015 | 34 | 30 | ns |
| **Treatment (%)** | | | | | | | | | | | | | |
| Prednisone 10 mg or more | 16.5 | 26 | 13.5 | 0.003 | 17 | 11 | ns | 12 | 18.5 | ns | 9.5 | 13.5 | ns |
| Warfarine | 14 | 10 | 15 | ns | 4.5 | 18 | 0.008 | 6 | 17 | 0.004 | 17 | 14 | ns |

| Proportions and significance | lower | p≤0.0005 | p≤0.005 | p<0.05 |  | p<0.05 | p≤0.005 | p≤0.0005 | higher |
| --- | --- | --- | --- | --- | --- | --- | --- | --- | --- |

The left column (all SLE) demonstrates distribution of investigated parameters for the whole SLE cohort. In the statistical analyses the subgroup of patients with high ((^H^), above 3^rd^ quartile) cytokine levels were compared to all the other patients. Statistical analyses of continuous variables were performed by Student t-test; values calculated as ratios were compared by non-parametric Mann-Whitney or Wilcoxon/Kruskal-Wallis test; all proportions were compared by 2 tailed Fishers exact test. *****- indicates definition by SLAM; **^☐^**- indicates definition by SLEDAI. List of abbreviations: aCL – anticardiolipin, B2GPI – beta 2 glycoprotein 1, LA-lupus anticoagulants, aPL - antiphospholipid abs, ESR – erythrocyte sedimentation rate, Hb - haemoglobin, WBC –white blood cells, PLT – platelets, U-alb/krea – urine albumin kreatinine ratio, p – p-value. Number of patients among the groups differ, since all IFN measurements were not available in all subjects due to technical reasons.
